# A New Species of *Gussevia* (Monopisthocotyla: Dactylogyridae) Parasitizing the Gills of *Astronotus Ocellatus* (Cichliformes: Cichlidae) in the Caatinga Domain, Northeastern Brazil

**DOI:** 10.1007/s11686-025-01210-z

**Published:** 2026-01-26

**Authors:** Ana Josilene Teles da Silva, Priscilla de Oliveira Fadel Yamada, Fabio Hideki Yamada

**Affiliations:** Laboratório de Ecologia Parasitária (LABEP), Universidade Regional do Cariri (URCA), Crato, Ceará 63105-000 Brazil

**Keywords:** Freshwater fish, Ectoparasite, Monogenea, Neotropical region, Taxonomy

## Abstract

**Purpose:**

A new species of *Gussevia* Kohn et Paperna, 1964 (Monopisthocotyla, Dactylogyridae) is described based on specimens recovered from the gills of *Astronotus ocellatus* (Agassiz, 1831) (Cichliformes, Cichlidae) collected in the Ubaldinho weir, state of Ceará, Brazil.

**Materials and methods:**

Some obtained monopisthocotylans were mounted on slides using Gray and Wess medium and studied using an optical microscope with a phase-contrast. Illustrations were made using a drawing tube attached to a Leica DM750 microscope and vectorized using Inkscape 1.4 software.

**Results:**

*Gussevia lamarckii* n. sp. is characterized by having an accessory piece nonarticulated with the MCO base, comprising two dorsal subunits, one Y-shaped and the other one rod-shaped; and an elongated peduncle. Furthermore, the new species can be easily distinguished from the other congeners by possessing (1) a ventral anchor with an external protuberance at the junction between the root and the shaft, and (2) a superficial groove at the base of the dorsal anchor.

**Conclusions:**

This study presents a new species of the genus *Gussevia*, the fourth described from *Astronotus* fish and the first from *A. ocellatus* and the aquatic ecosystems of the Caatinga domain.

## Introduction

The Neotropical region is distinguished by hosting the world’s largest and most diverse freshwater fish fauna, with the number of described species continues to rise [1]. The diversity of aquatic ecosystems promotes the diversification and evolution of parasites, such as monopisthocotylans of the family Dactylogyridae, which are ectoparasites that mainly infest the gills of teleost fish, interfering with their physiology and behavior [2].

*Astronotus ocellatus* (Agassiz, 1831), a freshwater cichlid (Cichliformes) commonly known as “apaiari” or “oscar” and native distributed to the Amazon River basin, encompassing Brazil, Peru, Colombia, and French Guiana [3, 4]. This medium-sized species has a wide distribution and high dispersal capacity and has been introduced into some river basins in northeastern and southern Brazil [5]. It also holds significant economic and commercial importance [3]. To date, 34 parasitic associations have been reported for *A. ocellatus*, belonging to: Myxozoa, Monopisthocotyla, Digenea, Cestoda, Nematoda, Acanthocephala, Mollusca, Hirudinea, Copepoda, and Branchiura (see Table 1).

Among the parasites of the class Monopisthocotyla, the genus *Gussevia* Kohn et Paperna, 1964 comprises species that parasitize the gills of cichlids [6]. Seventeen valid species of *Gussevia* are currently recognized (see Table 2), with 13 occurring in Brazil, and three described from *Astronotus crassipinnis* (Heckel, 1840) and *A. ocellatus* (Agassiz, 1831) from the Amazon River basin [7, 8]. In this study, during an investigation of the parasitic fauna of *A. ocellatus* in the Ubaldinho dam, Ceará state, Brazil, we describe a new species of *Gussevia*. Additionally, we report a new occurrence of *G. asota*, *G. astronoti*, and *G. rogersi*, which were found in the gills of this fish.

**Table 1:** Checklist of metazoan parasites of *Astronotus ocellatus* in Brazilian freshwater ecosystems

Parasite taxa	Locality	Reference
**Myxozoa**		
*Kudoa ocellatus* Silva, Silva, Lima, Matos, Sanches, Matos et Hamoy, 2022	Arari River Basin	[9]
**Monopisthocotyla**		
*Gussevia asota* Kritsky, Thatcher et Boeger, 1989	Guandu River Basin; Amazon Basin; Igarapé Fortaleza Basin; Guandu River Basin	[7, 10–13]
*Gussevia astronoti* Kritsky, Thatcher et Boeger, 1989	Amazon Basin	[7, 10 –11]
*Gussevia rogersi* Kritsky, Thatcher et Boeger, 1989	Amazon Basin	[7]
*Gussevia* sp.	Amazon Basin; Guandu River Basin	[5, 11]
**Digenea**		
*Clinostomum marginatum* (Rudolphi, 1819)	Igarapé Fortaleza Basin	[13]
*Herpetodiplostomum* sp.	Amazon Basin	[11, 14]
*Posthodiplostomum* sp.	Amazon Basin; Igarapé Fortaleza Basin	[11–15]
*Thometrema* sp.	Igarapé Fortaleza Basin	[13]
**Cestoda**		
*Proteocephalus gibsoni* Rego et Pavanelli, 1991	Igarapé Fortaleza Basin	[13]
*Proteocephalus* sp.	Amazon Basin	[15]
Unidentified cysts	Jaguaribe River Basin	[16]
**Nematoda**		
*Camallanus* sp.	Amazon Basin	[17]
*Contracaecum* sp. (larvae)	Guandu River Basin; Amazon Basin; Igarapé Fortaleza Basin; Jaguaribe River Basin	[5, 11, 13–18]
*Goezia spinulosa* (Diesing, 1839)	Amazon Basin	[17]
*Procamallanus (Spirocamallanus) inopinatus* Travassos, Artigas et Pereira, 1928	Amazon Basin	[12, 17]
*Procamallanus spiculastriatus* Pinheiro, Melo, Monks, Santos et Giese, 2018	Amazon Basin	[15]
*Pseudoproleptus* sp.	Amazon Basin	[15]
*Serpinema trispinosum* (larvae)	Jaguaribe River Basin	[16]
**Acanthocephala**		
Acanthocephala gen. sp.	Amazon Basin	[15]
*Polymorphus* sp.	Bacia do Rio Guandu	[5, 19]
**Mollusca**		
Bivalve fam. gen. sp.	Guandu River Basin	[5, 19]
**Hirudinea**		
Glossiphoniidae gen. sp.	Amazon Basin	[11]
*Placobdella* sp.	Guandu River Basin	[5, 19]
**Copepoda**		
*Ergasilus* sp.	Amazon Basin	[20]
*Lamproglena monodi* Capart, 1944	Guandu River Basin	[19]
*Lamproglena* sp.	Guandu River Basin	[5]
*Therodamas elongatus* (Thatcher, 1986)	Amazon Basin	[20]
**Branchiura**		
*Argulus* sp.	Amazon Basin	[21, 22]
*Argulus multicolor* Schuurmans Stekhoven J.H. Jr, 1937	Igarapé Fortaleza Basin	[13]
*Dolops bidentata* (Bouvier, 1899)	Amazon Basin	[21, 22]
*Dolops discoidalis* Bouvier, 1899	Amazon Basin	[21, 22]
*Dolops geayi* (Bouvier, 1897)	Amazon Basin	[21, 22]
*Dolops nana* Lemos de Castro, 1950	Amazon Basin	[11]

## Materials and Methods

Thirty-one specimens of *A. ocellatus* were collected in January 2023 from the Ubaldinho weir, Cedro municipality, state of Ceará, Brazil (6°35’08.04” S; 39°14’30.48” W). All procedures involving animals were conducted in accordance with the guidelines of the Animal Experimentation Ethics Committee (CEUA/URCA #00165/2018.1), and collection and transport were authorized by SISBIO (Biodiversity Authorization and Information System, authorization #61328-2). Hosts were captured using trawl nets and cast nets, individually stored in plastic bags, and subsequently frozen. Some gills from freshly killed host specimens were removed and fixed in heated alcohol. During necropsy, the gills were removed, placed in Petri dishes with water, and examined under a stereomicroscope to verify the presence of ectoparasites. Monopisthocotylans were mounted on slides using Gray and Wess medium [23] for the study of their structures and subsequent identification. In this study, no specimens were obtained for molecular analysis.

Morphology and morphometry of the parasites were analyzed using an optical microscope equipped with a computerized system for phase-contrast image analysis (Zeiss Axioscope 5). Illustrations were made using a drawing tube attached to a Leica DM750 microscope with phase-contrast optics and were vectorized using Inkscape 1.4 software. All measurements, expressed in micrometres (µm), represent straight-line distances between extreme points and are reported as the mean, followed by the range and the number (*n*) of specimens measured in parentheses, in accordance with the procedures of Kritsky et al. [7]. Direction of the male copulatory organ (MCO; counterclockwise vs. clockwise) followed Kritsky et al. [24]. Type specimens were deposited in the Helminthological Collection of the Instituto Oswaldo Cruz (CHIOC), state of Rio de Janeiro, Brazil. Other paratypes were deposited in the Helminthological Collection of the Institute of Parasitology České Budějovice, Czech Republic (IPCAS) and in the Helminthological Collection of the Institute of Biosciences (CHIBB), Botucatu, São Paulo State, Brazil.

## Results

Class Monopisthocotyla Brabec, Salomaki, Kolísko, Scholz et Kuchta, 2023.

Order Dactylogyridea Bychowsky, 1937.

Family Dactylogyridae Bychowsky, 1933.

*Gussevia* Kohn et Paperna, 1964.

**Fig. 1:**
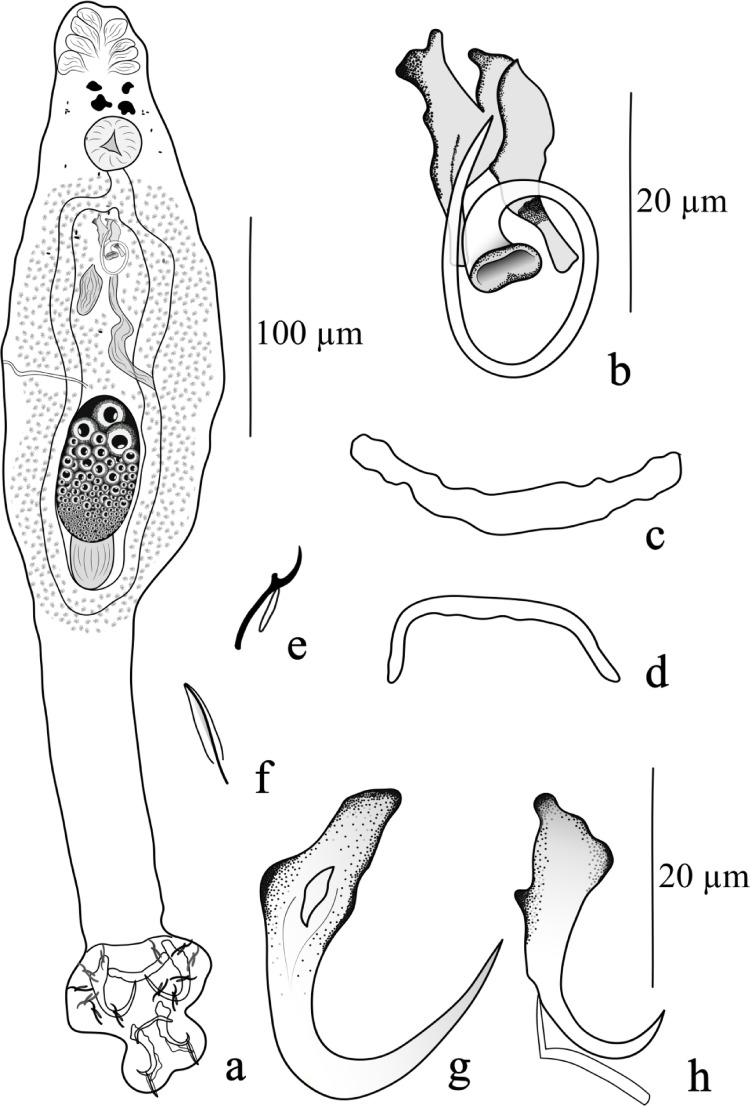
*Gussevia lamarckii* n. sp. **(a)** Holotype whole-mount (ventral); **(b)** MCO (ventral view); **(c)** Dorsal bar; **(d)** Ventral bar; **(e)** Hooks pairs 1–4 and 6–7; (**f**) Hook pair 5; (**g**) Dorsal anchor; (**h**) Ventral anchor

**Fig. 2:**
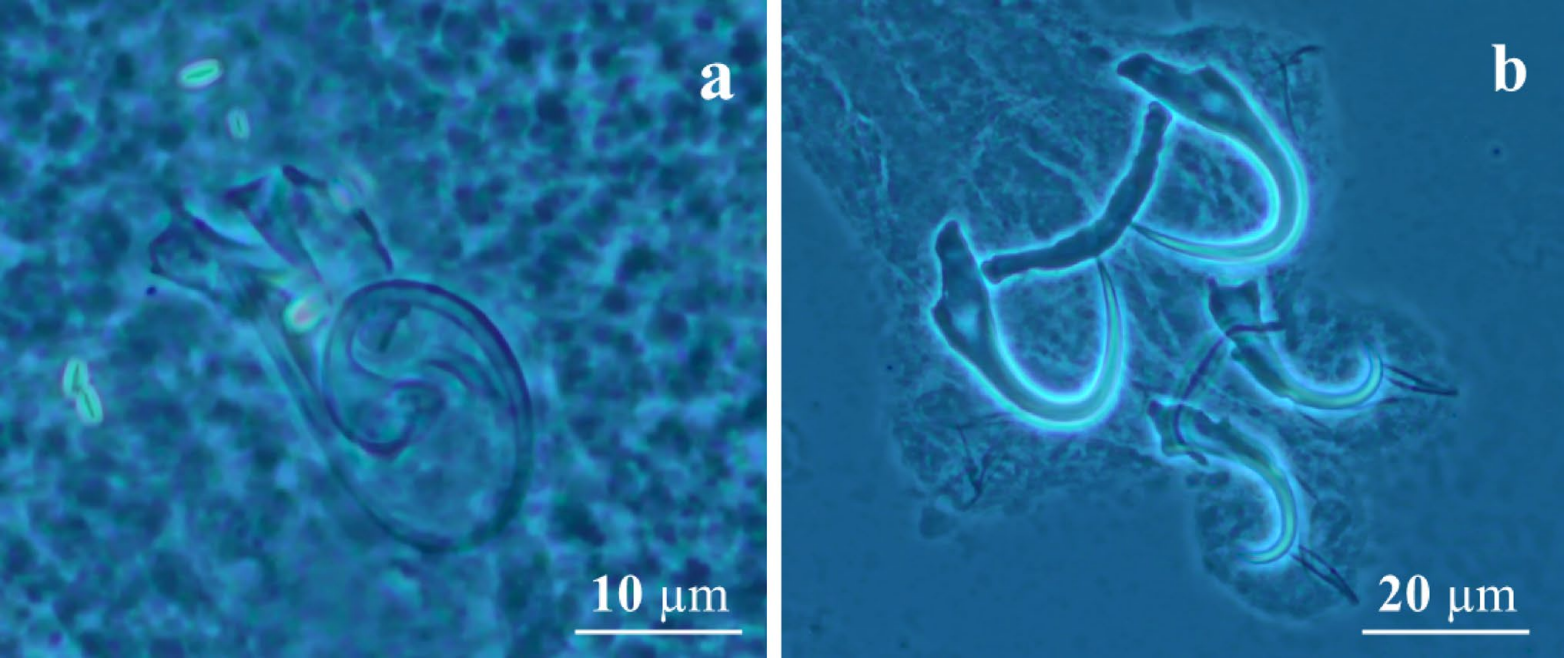
Photomicrographs of Holotype *Gussevia lamarckii* n. sp. (**a**) Copulatory complex; (**b**) haptor

*Gussevia lamarckii* n. sp.

(Figures 1a–h and 2 a–b)

**Description.** [Based on fifteen specimens]. Body elongated, fusiform, 524 (351–687; *n* = 15) long, 84 (42–123; *n* = 15) wide at mid-body. Cephalic region narrow; cephalic lobes moderately developed; four pairs of head organs; cephalic glands not observed. Four equidistant eyes; accessory granules elongated, scarce in the anterior trunk and cephalic region. Pharynx spherical 30 (17–58; *n* = 15) in diameter; esophagus short. Two intestinal caeca confluent and posterior to the gonads. Peduncle elongated. Haptor trapezoidal, 83 (52–179; *n* = 15) long, 84 (42–123; *n* = 15) wide. Ventral anchor 30 (27–32; *n* = 15) long, 10 (7–12; *n* = 15) wide, with subsquare base, with an external protuberance at the junction between the base and the shaft, deep root poorly differentiated, short anterior projection from tip of superficial root, curved shaft and acute point. Dorsal anchor 24 (20–27; *n* = 15) long, base 7 (6–8; *n* = 15) wide, with a superficial groove at the base, with well-developed superficial root, short deep root, curved shaft, and slightly tapered point. Ventral bar delicate, 30 (23–35; *n* = 15) long, slender; dorsal bar 18 (13–22; *n* = 15) long, yoked-shaped, with enlarged ends. Hook pairs 1–4, 6, and 7 similar in size, measuring 10 (8–12; *n* = 8) long; with slender shank, erect thumb, curved shaft, and delicate point; FH loop approximately ½ of shank length. Hook pair 5 delicate and filamentous; FH loop approximately ⅔ of shank length. Copulatory complex consists a male copulatory organ (MCO) and a sclerotized accessory piece. MCO composed by a clockwise ring, 18 (16–22; *n* = 15) long. Accessory piece, 17 (16–22; *n* = 15) long, non-articulated with the MCO base, comprising two dorsal subunits, one Y-shaped and the other one rod-shaped. Gonads intercaecal; ovary elongate, 55 (45–73; *n* = 4) long, 34 (29–38; *n* = 4) wide; testis 32 (24–39; *n* = 4) long, 22 (13–36; *n* = 4) wide, dorsal to ovary. Vas deferens looping left intestinal caecum, dilating to form an elongate seminal vesicle. Single prostatic reservoir. Vagina dextral, consisting of a slightly sclerotized tube opening near mid-body. Vitellaria dense, absent near reproductive organs. Oviduct, ootype, uterus, seminal receptacle, and eggs not observed.

## Taxonomic Summary

**Type host**: *Astronotus ocellatus* (Agassiz, 1831) (Cichliformes: Cichlidae).

**Type Locality**: Ubaldinho weir, Salgado River basin, municipality of Cedro, state of Ceará, Brazil (6°35’08.04” S; 39°14’30.48” W).

**Infestation Site**: Gills.

**Infestation parameters**: Seven infested out of 31 analyzed hosts; prevalence: 22.5%; total number of parasites: 15; mean intensity: 2.14 ± 0.55.

**Type Specimens**: Holotype CHIOC 40887; Paratypes CHIOC 40888, 40889, 40890; IPCAS M-844 and CHIBB 940 L, 941L, 942L, 943L, 944L, 945L, 946L, 947L, 948L.

**ZooBank registration**: (urn: lsid: zoobankACD4056A-F697-4DF0-9403-17F37EB3FD4F).

**Etymology**: The specific epithet *lamarckii* refers to the morphology of the elongated peduncle and in allusion to the long neck, emblematic of Lamarckism, recalling Lamarck’s theory of evolutionary transformation.

## Remarks

The new species was assigned to the genus *Gussevia* revised by Kritsky et al. [6] mainly by possessing a haptor with two lobes (anterior and posterior); a modified ventral anchor possessing a conspicuous anchor filament; a hook pair 5 modified, usually delicate and lying on the posterior haptor lobe; and a copulatory complex consisting of nonarticulated MCO and accessory piece. *Gussevia lamarckii* n. sp. resembles *G*. *rogersi* Kritsky, Thatcher et Boeger, 1986 and *G. asota* Kritsky, Thatcher et Boeger, 1986 by having a MCO with a coil of about 1 to 1½ rings; and a slightly thickened ventral bar and a broadly U or V-shaped dorsal bar. However, the new species differs from *G. rogersi* and *G. asota* by possessing an accessory piece nonarticulated with the base of the MCO, composed of two dorsal subunits, one Y-shaped and the other rod-shaped, and an elongated peduncle. Furthermore, the new species can be easily distinguished from the other congeners by possessing (1) a ventral anchor with an external protuberance at the junction between the root and the shaft, and (2) a superficial groove at the base of the dorsal anchor.

## Discussion

In the Neotropical region, monopisthocotylans parasites of cichlids have been recorded in various aquatic ecosystems of South America, Central America, and Mexico, highlighting their wide occurrence across the native distribution areas of cichlid hosts [25–28]. It comprises species of *Trinidactylus* Hanek, Molnar et Fernando, 1974; *Gussevia* Kohn et Paperna, 1964; *Sciadicleithrum* Kritsky, Thatcher et Boeger, 1989; *Tucunarella* Mendoza-Franco, Scholz et Rozkošná, 2010; *Parasciadicleithrum* Mendoza-Palmero, Blasco-Costa, Hernández-Mena et Pérez-Ponce de León, 2017; and *Biotodomella* Morey, Arimuya et Boeger, 2019 [26–28]. Kritsky et al. [6] pointed out that members of the genus *Gussevia* can be distinguished from other dactylogyrids by the combined presence of (1) overlapping gonads; (2) a haptor with anterior and posterior lobes; (3) a modified ventral anchor with a well-developed filament; (4) hook pair 5 modified, usually delicate and located on the posterior haptoral lobe together with the ventral anchors; and (5) a coiled cirrus with clockwise rings. The new species described herein represents the fourth member of the genus *Gussevia* reported from the genus *Astronotus* (Table 2).

**Table 2:** Species of *Gussevia* Kohn et Paperna, 1964 (Monopisthocotyla: Dactylogyridae) from Neotropical region described to date. Scientific names of host species are given according to accepted names in Froese et Pauly [4]

Gussevia species	MCO*	Host species	Locality	Reference
*G. spiralocirra* Kohn et Paperna, 1964 (type specie)	NA	*Pterophyllum scalare*	Atacuari River, Peru	[29]
*G. alii* (Molnar, Hanek et Fernando, 1974)	NA	*Cichlasoma bimaculatum*	Talparo River, Trinidad	[6]
*G. alioides* Kritsky, Thatcher et Boeger, 1986	NA	*Heros severus*	Solimões River, Amazonas, Brazil	[6]
*G. arilla* Kritsky, Thatcher et Boeger, 1986	NA	*Cichla ocellaris*	Negro River, Amazonas, Brazil	[6]
*G. asota* Kritsky, Thatcher et Boeger, 1989	A	*Astronotus crassipinnis*,* Astronotus ocellatus*	Janauacá Lake, Amazonas, Brazil	[7]
*G. astronoti* Kritsky, Thatcher et Boeger, 1989	A	*Astronotus crassipinnis*,* Astronotus ocellatus*	Janauacá Lake, Amazonas, Brazil	[7]
*G. cichlasomatis* (Molnar, Hanek et Fernando, 1974)	NA	*Cichlasoma bimaculatum*	Nariva swamp, Trinidad	[6]
*G. dispar* Kritsky, Thatcher et Boeger, 1986	NA	*Heros severus*	Solimões River, Amazonas, Brazil	[6]
*G. disparoides* Kritsky, Thatcher et Boeger, 1986	NA	*Heros severus*	Solimões River, Amazonas, Brazil	[6]
*G. dobosi* (Molnar, Hanek et Fernando, 1974)	NA	*Cichlasoma bimaculatum*	Nariva swamp, Trinidad.	[6]
*G. elephus* Kritsky, Thatcher et Boeger, 1986	NA	*Uaru amphiacanthoides*	Negro River, Amazonas, Brazil	[6]
*G. herotilapiae* Vidal-Martinez, Scholz et Aguirre-Macedo, 2001	NA	*Herotilapia multispinosa*	Mahogany River, Nicaragua	[25]
*G. longihaptor* (Mizelle et Kritsky, 1969)	NA	*Cichla ocellaris*	Amazon River, Brazil	[6]
*G. obtusa* Kritsky, Thatcher et Boeger, 1986	NA	*Uaru amphiacanthoides*	Negro River, Amazonas, Brazil	[6]
*G. lamarckii* n. sp.	NA	*Astronotus ocellatus*	Ubaldinho weir, Ceará, Brazil	
*G. rogersi* Kritsky, Thatcher et Boeger, 1989	A	*Astronotus crassipinnis*,* Astronotus ocellatus*	Solimões River, Amazonas, Brazil	[7]
*G. tucunarense* Kritsky, Thatcher et Boeger, 1986	NA	*Cichla ocellaris*	Negro River, Amazonas, Brazil	[6]
*G. undulata* Kritsky, Thatcher et Boeger, 1986	NA	*Cichla ocellaris*	Negro River, Amazonas, Brazil	[6]

*Accessory piece articulated with the MCO base (A) or non-articulated with the MCO base (NA)

This study provides the first record of the genus *Gussevia* parasitizing a fish host in northeastern Brazil, specifically in the state of Ceará. Despite recent advances in parasitological research across Brazil, the ichthyoparasitic fauna of the semiarid northeastern Brazil remain limited. This region harbors a rich and diverse fish fauna, including both native and introduced species adapted to extreme environmental conditions, which may serve as valuable hosts for new or poorly known parasite species. Therefore, documenting parasite diversity in these aquatic ecosystems is essential to improve our understanding of host–parasite relationships and the biogeography of Neotropical monopisthocotylans [16, 30–34]. The findings of this study expand current knowledge of the parasitic fauna of fishes in the Caatinga biome, underscoring the potential for the discovery of additional species in this environment.

## Data Availability

No datasets were generated or analysed during the current study.
